# Consensus statement on the neurocognitive outcomes for early detection of mild cognitive impairment and Alzheimer dementia from the Chinese Neuropsychological Normative (CN-NORM) Project

**DOI:** 10.7189/jogh.09.020320

**Published:** 2019-12

**Authors:** Huali Wang, Zili Fan, Chuan Shi, Lingchuan Xiong, Haifeng Zhang, Tao Li, Yongan Sun, Qihao Guo, Yanghua Tian, Qiumin Qu, Nan Zhang, Zaohuo Cheng, Liyong Wu, Daxing Wu, Zaizhu Han, Jinzhou Tian, Hengge Xie, Shuping Tan, Jingfang Gao, Benyan Luo, Xiaoping Pan, Guoping Peng, Bin Qin, Yi Tang, Kai Wang, Tao Wang, Junjian Zhang, Qianhua Zhao, Serge Gauthier, Xin Yu

**Affiliations:** 1 Dementia Care & Research Center, Peking University Institute of Mental Health (Sixth Hospital), Beijing, China; 2 Beijing Dementia Key Lab, Beijing, China; 3 National Clinical Research Center for Mental Disorders, Key Laboratory for Mental Health, National Health Commission, Beijing, China; 4 Department of Clinical Psychological Assessment, Peking University Institute of Mental Health (Sixth Hospital), Beijing, China; 5 Department of Neurology, Peking University First Hospital, Beijing, China; 6 Department of Geriatrics, Shanghai Sixth Hospital, Shanghai, China; 7 Department of Neurology, the First Affiliated Hospital of Anhui Medical University, Hefei, China; 8 Department of Neurology, the First Affiliated Hospital of Xi’an Jiaotong University, Xi’an, China; 9 Department of Neurology, General Hospital of Tianjin Medical University, Tianjin, China; 10 Wuxi Mental Health Center, Nanjing Medical University, Wuxi, China; 11 Department of Neurology, Xuanwu Hospital, Capital Medical University, Beijing, China; 12 Medical Psychological Center, The Second Xiangya Hospital, Central South University, Changsha, China; 13 State Key Laboratory of Cognitive Neuroscience and Learning & IDG/McGovern Institute for Brain Research, Beijing Normal University, Beijing, China; 14 Beijing Dongzhimen Hospital, Beijing University of Chinese Medicine, Beijing, China; 15 Department of Neurology, China PLA General Hospital, Beijing, China; 16 Beijing Huilongguan Hospital, Beijing, China; 17 Zhejiang University of Traditional Chinese Medicine First Affiliated Hospital, Hangzhou, China; 18 Department of Neurology, First Affiliated Hospital, Zhejiang University School of Medicine, Hangzhou, China; 19 Guangzhou First People's Hospital, School of Medicine, South China University of Technology, Guangzhou, China; 20 Beijing Hospital, National Health Commission, Beijing, China; 21 Shanghai Mental Health Center, Shanghai Jiaotong University School of Medicine, Shanghai, China; 22 Department of Neurology, Zhongnan Hospital, Wuhan University, Wuhan, China; 23 Department of Neurology, Huashan Hospital, Fudan University, Shanghai, China; 24 McGill Center for Studies in Aging, McGill University, Montreal, Canada

**Figure:**
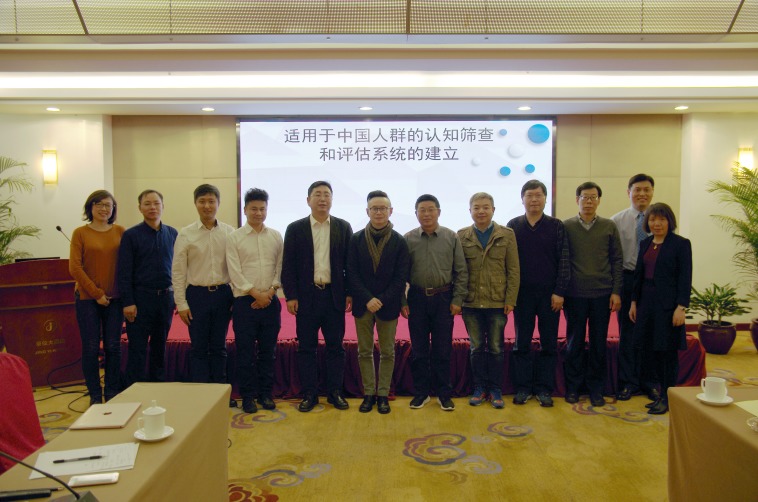
Photo: The group photo of expert panel at the consensus meeting on November 30, 2018 (from Huali Wang’s own collection, used with permission).

It was recently estimated that 10.4 million individuals are living with dementia in China [1]. However, under-recognition of dementia and related cognitive impairment remains a substantial challenge and thus results in a delay in seeking appropriate care as early as possible [2]. The misbelief that dementia and mild cognitive impairment are natural processes of normal aging has prevented families from seeking a timely diagnosis [3,4]. Moreover, clinicians often encounter difficulties in distinguishing dementia and mild cognitive impairment (MCI) from normal age-related cognitive change because many cognitive abilities naturally decline as individuals become older [5]. Therefore, an accurate neuropsychological assessment is a critical step to assist clinicians or researchers in the early detection of dementia and mild cognitive impairment.

Fundamentally, we need a set of sensitive and specific cognitive assessment instruments to detect subtle cognitive changes present in the early phase of neurocognitive disorders that may be missed during a routine clinical evaluation. Currently, most neurocognitive tests are developed by English-speaking researchers. To assess cognitive impairment in non-English-speaking populations, researchers have developed new tools or translated and adapted tests initially developed for English-speaking individuals. The newly developed tools may be more sensitive to linguistic and cultural differences [6]. The validation process of these tools is often complex, time-consuming and expensive. Most researchers prefer translating and validating the instruments from English to the local language, such as Chinese, Spanish and other languages. Therefore, lack of a harmonized protocol for neurocognitive assessment is a great challenge for researches on neurocognitive disorders.

The screening instruments, such as the Mini-Mental State Examination (MMSE) [7], cognitive ability screening instrument (CASI) [8], and Montreal Cognitive Assessment (MoCA) [9], and the neuropsychological battery tests, such as the Alzheimer Disease Assessment Scale-Cognitive portion (ADAS-Cog) [10] and neuropsychological test battery (NTB) [6], have been translated and adapted in Chinese. However, the validity of these instruments among Chinese elders is affected by a complex network of social and psychological factors. These factors include a lack of literacy, culture-specific factors related to individual test items (linguistic differences), and the overall lack of normative test data for the population.

Literacy, which consists of the ability to read and write, may be reflected in the neuropsychological performance. Nearly 37.4 million Chinese individuals aged 60 and older are illiterate. Previous studies have shown that low education is associated with a higher risk of cognitive impairment [11]. The lower cognitive reserve due to less lifetime exposure to rich educational experiences might explain this association [12]. However, the associative dependence of existing instruments on education may hinder a culturally fair evaluation. Therefore, the poor performance on cognitive tests for illiteracy should be interpreted with substantial caution. Educational attainment was significantly associated with the score of an intelligence test, particularly fluid intelligence [13]. Moreover, schooling may also have an impact on the neuropsychological test performance relevant to diverse abilities, including memory, language, problem-solving, constructional abilities, and calculation abilities [14,15].

Several studies have proven the effect of education on the performance on various neurocognitive measures, such as the test of the MoCA [16,17], MMSE [7], and language test [15,18]. Therefore, further analysis of illiteracy may provide a better understanding of brain function in elderly individuals. For example, adjustment, eg, adding points to the illiterate population, should be considered in adopting the scoring rules of the translated cognitive tests. Otherwise, the prevalence of cognitive impairment may be overestimated using the cut-off score proposed in the original paper conducted in the English-speaking population.

In contrast with the verbal intelligence and reading test, the performance intelligence and nonreading test, eg, the Wisconsin Card Sorting Test (WCST), is less sensitive to education. It implies that tests that tap into the performance intelligence and everyday problem solving may provide a more culturally fair evaluation of cognitive function for illiterate individuals.

The paucity of culturally and linguistically appropriate neuropsychological measures constitutes another significant barrier to the identification of dementia. In terms of cultural-specific factors and linguistic differences, the availability and complexity of words within a language may make translating and adapting existing tests challenging. For example, in the Trail making test B ((TMT-B), the subject is instructed to connect the letters and numbers alternatively. Many of the current cohorts of Chinese elders never had the opportunity to learn English; thus, they cannot complete the TMT-B. Therefore, several alternative forms of the TMT-B have been developed for Chinese individuals, eg, the shape TMT, color TMT, or black and white TMT [19-22].

Another example is the FAS test to measure phonetic fluency. Chinese elders cannot produce words that start with the letters “F,” “A” and “S.” Some researchers have proposed asking the subject to produce words that start with the Chinese character starting with “fa (发),” “shui (水),” and “kai (开)” [23]. However, the score of the test was typically very low in the elderly individuals regardless of their cognitive status. Thus, this type of test is not appropriate for Chinese elders.

Last, but also important, the insufficient normative data impede the application of existing neurocognitive tests, particularly in the under-representative populations, including individuals living in remote areas. A neuropsychological assessment incorporates the administration of validated and standardized psychometric tests to assess intelligence, memory and other cognitive abilities. Comparison of an individual’s test scores to a normative reference group indicates whether the performance is at the expected level or impaired [24]. The normative data are particularly critical for the definition of subjective cognitive decline. Moreover, there is a strong demand for the development of digital cognitive testing due to a lack of professional clinical psychologists responsible for administering and interpreting the neuropsychological tests, particularly in underrepresented settings. Appropriate normative data matched to the characteristics of the individual being assessed are essential for valid interpretation of their test performance and to maximize the diagnostic accuracy. The normative data based on Caucasian elders cannot be generalized to Chinese elders and participants with lower educational levels. Several instruments included in the MATRICS Consensus Cognitive Battery (MCCB), such as the HVLT and trail making tests, only have normative data for individuals aged from 20-59 years old [25]. The normative data for Chinese elders have not been developed.

To confront the previously described cross-cultural neuropsychological research challenges, the Ministry of Science and Technology of China has funded the Chinese Neuropsychological Normative Project (CN-NORM), which aims to reach a consensus on cognitive tests relevant for Chinese population, and to establish the Normative reference of the neuropsychological instruments that are applicable for measuring the outcome of mild cognitive impairment and Alzheimer dementia. Therefore, the CN-NORM project includes three stages:

Stage I: selecting the potential useful neuropsychological instruments for the detection of mild cognitive impairment and Alzheimer disease (AD) in Chinese individuals;

Stage II: establishing the norms in a nationally representative sample, taking age, education, gender, household registry (ie, *hukou* in Chinese), and geographic distribution into consideration;

Stage III: validating the norms in clinical samples (ie, mild cognitive impairment, Alzheimer disease, frontotemporal dementia, dementia of Lewy body, vascular dementia, late-life depression and schizophrenia) to explore the optimal neuropsychological measures for the detection of MCI and subtypes of dementias.

Herein, we reported the consensus statement that resulted from the expert survey and consensus meetings at Stage I. We anticipate the consensus statement will not only guide the development of a normative reference for these selected tests for Chinese population, but will also inform future clinical research on the continuum of Alzheimer disease by providing culturally appropriate neuropsychological test instruments.

## THE SCIENTIFIC ADVISORY GROUP (SAG)

The core group members are X.Y., H.W., C.S., and Y.S. X.Y. is the principal investigator for the CN-NORM project. H.W. is the coordinator providing linkage between the Scientific Advisory Group, the expert panel, and the project team.

The SAG members and expert panelists were recruited per their academic qualifications in neuropsychology, expertise in dementia research, particularly on neuropsychological studies, and experience in developing the normative data of neurocognitive tests.

### Four phases of the working program

**Figure 1:**
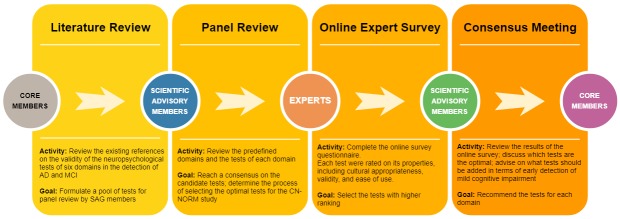
Flow diagram for reaching the consensus on the neurocognitive outcomes.

The process used to reach a consensus included four phases: literature review, panel review, online expert survey, and consensus meeting (**Figure 1**).

#### Phase I. Literature review

The references that reviewed or studied the utility of neurocognitive tests in discriminating Alzheimer disease from other diagnostic entities or healthy controls were searched and reviewed. The tests tap into six domains as defined in the chapter of *neurocognitive disorders* by the DSM-5, including complex attention, learning and memory, executive function, language, psychomotor perceptual, and social cognition. A three-step strategy was used to search the literature (refer to details in Appendix S1 in **Online Supplementary Document**).

#### Phase II. Panel review to determine the neuropsychological tests for the expert survey

By reference to the literature review, we compared the relative discriminability and psychometric properties of the neuropsychological tests. We subsequently determined which neuropsychological tests might be optimal to demonstrate changes in six cognitive domains in MCI and early AD according to the following criteria:

- Validity concerning the different levels of cognitive impairment, particularly for the discrimination between MCI and normal controls
- Availability of a Chinese version
- Psychometric properties and cross-cultural suitability in Chinese individuals

Four SAG members and four core members convened on Sept. 4, 2018 to review the appropriateness of the cognitive domains and proposed the neurocognitive tests for the expert survey.

#### Phase III. Expert survey

The request to complete the online survey questionnaire (https://www.wjx.top/jq/31231718.aspx) was sent to the expert panelists. Thirty-one panelists responded and completed the survey. The response rate was 83.8%.

The online survey provided the essential information of the proposed tests, including the name of the test, the domain the test measures, the year of development, the brief instructions on to administer the test, the primary measures, and the duration of the test. Moreover, it also introduced the utility (eg, sensitivity and specificity) of the test in the detection of AD and MCI.

The experts were first instructed to provide a self-rating score of his/her familiarity with the test (Cs, range from 0 ~ 9, a higher score indicated more familiarity with the test) and then scored the properties of the candidate tools (Table S1 in **Online Supplementary Document**). The expert subsequently decided whether the test could be used as the major neurocognitive outcome of AD and MCI [ranged from 0 (not needed) ~ 8 (mostly required)]. Moreover, he/she used a 7-level Lite scale to rate the evidence for making the expert judgement (Ca, meta-analysis or systematic review = 1.00, peer-review papers published in international journals = 0.80, peer-review papers published only in Chinese = 0.60, practical experiences = 0.40, the information provided in the questionnaire = 0.20, and personal intuition = 0).

To select the most situationally appropriate tests, the criteria in our study for reaching the consensus included that the statement achieved a median score of 7.00 or higher, the interquartile range (IQR) ≤ 2.0 and the coefficient of variation (CV) < 0.3 [26,27]. An authoritative coefﬁcient (Cr), composed of evidence in making an expert judgment (Ca) and the familiarity with the instrument (Cs), greater than 0.7 is acceptable [28].

#### Phase IV. Making recommendations on the consensus meeting

Eight SAG members who did not attend the first SAG meeting and four core members convened on November 30, 2018. During the consensus meeting, the results of the online expert survey, including the statistics and the narrative comments, were presented. The following questions were extensively discussed:

- What is the minimum number of tests to be selected for each domain?
- How should we address the necessity to include the tests that do not reach an acceptable level during the online survey, eg, the memory binding test or the four mountains test?
- Which language tests are potentially culturally fair and easy to administer?
- Should we introduce the Flanker test into the neurocognitive outcome as it is free of an education effect?
- Are the tests on visuospatial function sufficient to measure the psychomotor perceptual function?
- What is the additional value of social cognition in the detection of AD and MCI?
- How should we deal with the tests that could tap into two or more cognitive domains?

## QUALITATIVE REVIEW OF THE LITERATURE

As summarized in **Table 1**, the literature has shown that the cognitive profiles of AD and MCI are different from other types of dementia (eg, VaD, FTD and DLB) and other psychiatric disorders in old age (eg, schizophrenia and major depressive disorder).

**Table 1:** The relative deficit of cognitive domains in different types of neuropsychiatric disorders

Cognitive domain	MCI	Mild to moderate AD	VaD	FTD	DLB	MDD
Attention	TBD	†	†	†	‡	‡
Memory	†	‡	TBD	†	†	TBD
Executive Function	TBD	‡	‡	‡	‡	†
Language	TBD	†	TBD	‡	TBD	TBD
Visuospatial	TBD	‡	*	-	‡	*
Social Cognition	TBD	†	TBD	‡	TBD	TBD

TBD – to be determined, AD – Alzheimer disease, MCI -ild cognitive impairment, VaD – vascular dementia, FTD – frontotemporal dementia, DLB – Lewy body dementia, MDD – major depressive disorder (in old age)

*Subtle deficit.

†Mild impairment.

‡Significant impairment.

Each cognitive test has a different utility in discriminating MCI and AD from healthy controls, as well as from other types of dementia. Briefly, the memory domain was most studied, and the social cognition domain was least explored. Domains of attention, executive function, visuospatial function, and language have not previously been extensively explored for the detection of MCI. Refer to additional details in Appendix S2 and Table S2 in **Online Supplementary Document**.

## DETERMINING THE INSTRUMENTS TO BE INCLUDED FOR THE EXPERT STUDY

At the first panel review meeting, the experts recommended 4-8 tests for the assessment of each domain as follows: four tests for attention, five tests each for executive function, language, and social cognition, seven tests for memory, and eight tests for visuospatial function (the list shown in **Table 2**).

**Table 2:** The statistics of the online expert survey

	Priority rating					Expert self-evaluation		
	**Mean**	**SD**	**Median**	**CV**	**IQR**	**Cs**	**Ca**	Cr*
**Attention:**								
Flanker test	4.133	2.46	5	59.5%	4.25	0.34	0.48	0.42
Digit-symbol substitute test	6.774	1.359	7	20.1%	2	0.85	0.61	0.74
Trail making test – A (TMT-A)	7	1.033	7	14.8%	2	0.96	0.65	0.80
Choice reaction time	4.4	2.401	5	54.6%	3.25	0.45	0.52	0.49
**Memory:**								
Hopkins verbal learning test (HVLT)	5.71	1.987	6	34.8%	3	0.71	0.60	0.65
Memory binding test (MBT)	4.9	1.954	5	39.9%	2	0.29	0.41	0.35
California verbal learning test (CVLT)	5.6	1.886	6	33.7%	3	0.70	0.62	0.66
Rey auditory verbal learning test (RAVLT)	5.387	2.092	5	38.8%	3	0.62	0.52	0.57
Free and cued selective reminding test (FCSRT)	5.533	2.03	6	36.7%	2	0.44	0.52	0.49
Repeatable battery for the assessment of neuropsychological status (RBANS) – story delayed memory	5.161	1.899	5	36.8%	2	0.46	0.50	0.48
Wechsler memory scale – logic memory	5.533	2.161	6	39.1%	3	0.60	0.56	0.58
**Executive function:**								
Stroop test	6.839	1.53	7	22.4%	2	0.93	0.59	0.76
Wisconsin card sorting test (WCST)	5.258	1.983	6	37.7%	1	0.74	0.62	0.68
Trail making test – B (TMT-B)	6.968	1.224	7	17.6%	2	0.92	0.66	0.79
Digit span	7.065	1.263	8	17.9%	2	0.95	0.66	0.81
Spatial span	4.467	2.063	4.5	46.2%	3.25	0.40	0.42	0.42
**Language:**								
Animal naming	6.548	1.41	7	21.5%	3	0.97	0.66	0.81
Western aphasia battery (WAB) – comprehension	4	2.101	4	52.5%	3.25	0.39	0.48	0.43
RBANS picture naming	4.067	2.333	4	57.4%	3.25	0.41	0.48	0.45
Boston naming test	6.419	2.029	7	31.6%	2	0.75	0.59	0.67
Phonic fluency test	5.065	2.065	5	40.8%	3	0.75	0.52	0.64
**Visuospatial function:**								
Four mountains test	3.452	1.981	4	57.4%	3	0.14	0.32	0.24
Supermarket trolley virtual reality	4.133	2.446	5	59.2%	4	0.18	0.31	0.25
Brief Visual Memory Test (BVMT)	4.935	2.159	5	43.8%	3	0.50	0.50	0.51
Line orientation test	4.516	2.365	5	52.4%	3	0.46	0.48	0.48
Clock drawing test	6.839	1.53	7	22.4%	2	0.97	0.68	0.83
Rey-Osterrieth complex figure test (ROCF)	5.276	1.98	6	37.5%	3	0.63	0.51	0.58
Block design	4.833	2.036	5	42.1%	3	0.63	0.51	0.57
CERAD constructional praxis	4.467	2.047	4	45.8%	3	0.35	0.43	0.39
**Social cognition:**								
Iowa gambling task (IGT)	4.767	2.473	5	51.9%	4	0.47	0.43	0.47
Game of dice task (GDT)	4.1	2.249	5	54.9%	3	0.31	0.30	0.31
Facial expression recognition test	5.71	2.283	6	40.0%	2	0.56	0.52	0.54
Eye emotional recognition task	4.806	2.301	5	47.9%	3	0.41	0.40	0.41
Faux pas recognition	3.833	1.931	4	50.4%	2	0.40	0.38	0.40

SD – standard deviation, CV – coefficient of variation, IQR – interquartile range, Cs – familiarity with the instrument, Ca – basis for expert judgment, Cr – authoritative coefﬁcient

*The inconsistence between mean Cr and the average of mean Cs and Ca was due to missing data in the certain tests.

## QUANTITATIVE RESULTS OF THE EXPERT SURVEY

As shown in **Table 2**, two tests of attention (ie, the digit-symbol substitute test and trail making test - A), three tests of executive function (ie, the Stroop test, trail making test – B, and digit span - backward) and one test of visuospatial function (ie, the clock drawing test) were consistently considered to be the appropriate neurocognitive outcome of AD and MCI (all CV<0.3, IQR ≤2). The animal naming test for measuring language was marginally in agreement (CV = 0.2, IQR = 3). The experts also showed a higher authority in making the judgment (an authoritative coefficient greater than 0.70) of the previously described tests, accompanied by a high familiarity with these tests and made their judgment based on higher-ranking references.

In contrast, there was a more significant variation in selecting the tests for measuring the cognitive domains, including memory, language and social cognition (CV > 0.3, IQR > 2). With the exception of the conventional tests, eg, the verbal learning test, logic memory, block design, Rey-Osterrieth complex figure test and Boston naming test, the experts were not familiar with the other recently introduced tests relevant to memory (eg, memory binding test, FCSRT, and RBANS), visuospatial function (eg, Four mountains test and supermarket trolley virtual reality), language (eg, WAB-comprehension and phonic fluency) and social cognition (eg, IGT, GDT, facial expression recognition task, and faux pas recognition). Most experts made their recommendation based primarily on the information provided in the questionnaire.

## RECOMMENDATIONS BY THE EXPERT PANEL OF THE CONSENSUS MEETING

At the consensus meeting, the experts agreed that for each domain, at least two tests should be selected to ensure a reliable assessment. For the tests that were not familiar to the expert panel, the tests were selected based on the literature review.

Furthermore, the experts reached a consensus that the tests should not only be sensitive for dementia but also, more critically, be sensitive for MCI screening. Moreover, the experts emphasized that the tests should not only support screening from the general population but also provide clues for the differential diagnosis between different types of cognitive disorders and between Alzheimer disease and other common psychiatric disorders in old age.

The experts also reviewed the specific domain that each test primarily measures. The digit symbol substitute test was moved from the domain of attention to executive function. The forward digit span task was categorized as a test for attention, while the backward digit span task reflected executive function.

The experts agreed that it was necessary to include the recently developed tests that have greater potential to discriminate MCI from normal elderly individuals. The tests that have been validated in Chinese, such as the memory binding test, will be prioritized. The tests that have not been validated in Chinese will not be included, such as the Four mountains test.

Finally, the experts proposed three tests for attention, four tests for memory, four tests for executive function, two tests for language, three tests for visuospatial function and three tests for social cognition (**Table 3**). These tests formed the CN-NORM Consensus Battery (CNCB). As described in Appendix S3 in **Online Supplementary Document**, most tests in the CNCB have been adapted and validated in Chinese, while several newly developed instruments will be adapted and validated in Chinese culture soon.

**Table 3:** The tests in the CN-NORM Consensus Battery

Domain	Cognitive test
Attention	Digit span – forward*
	Trail making test – A†
	Flanker test
Memory	Hopkin’s verbal learning test
	Brief visual memory test
	Logic memory
	Memory binding test
Executive function	Trail making test – B†
	Digit span – backward*
	Digit symbol substitution
	Stroop test
Language	Verbal fluency – animal naming
	Boston naming test
Visuospatial function	Clock drawing test
	Judgment of Line Orientation Test
	Visual object and space perception (VOSP) – Silhouettes
Social cognition	Facial emotion recognition test
	Iowa gambling task
	Game of dice task

*Subtest of the digit span test.

†Subtest of trail making test.

## CONCLUSIONS

Similar to the developing process of the MATRICS Consensus Cognitive Battery (MCCB) [29], the SAG of the CN-NORM project selected 17 tests (19 subtests) to form the beta-version of the CN-NORM Consensus Battery (CNCB) via a panel review, an expert survey and a consensus meeting. The CNCB tests cover six subdomains, including attention, memory, executive function, language, visuospatial and social cognition.

There are several criteria to select subtests. First, the subtest has been translated into Chinese and widely used in China, particularly for the verbal and language test. Second, it is easy to administer and the performance time is relatively short. Third, the stimulus materials are presented by figures and symbols as much as possible with the exception of the verbal and language test to minimize the cultural differences and adapt to illiterate subjects. Fourth, the reliability and validity of the subtest are established; there is evidence that it can distinguish normal aging individuals from MCI, MCI from dementia, or a different type of dementia, or dementia from other mental illness. Fifth, the subtest can be repeatedly used, and the practice effects are relatively small. Sixth, the subtest is easy to transfer to a computerized version and is therefore easy to use in a community hospital.

As expected, there are substantial commonalities in measuring attention, language, executive function and visuospatial function between our study and the ongoing dementia prevention trials in the US and European countries [30-32]. For these domains, we select at least two subtests for neurocognitive disorders. The tests for these cognitive domains, eg, the digit span, trail making test, verbal fluency, and Stroop test, are widely reported in dementia studies, such as the worldwide Alzheimer Disease Neuroimaging Initiative (WW-ADNI) [33]. Executive function includes very complicated processes, such as planning and mental flexibility [34,35]. Executive dysfunction is more prominent in vascular dementia, frontotemporal dementia, and major depressive disorder [36-39]. To facilitate the differential diagnosis of dementia, we intentionally include four subtests to increase its weight in the neuropsychological assessment.

As noted, there was some disagreement on selecting the memory tests in the expert survey. The comments and discussion at the face-to-face advisory meeting played a major role in reaching consensus when the experts considered the appropriateness, usefulness and innovation of each test as a whole. Similar to previous studies, we select the Hopkins verbal learning test and logic memory test in the proposed CNCB [30-32]. These two tests have been widely used to measure immediate and delayed recall of AD and aMCI. The decline in the delayed recall is considered a sign of impairment in episodic memory in typical AD [40-43]. However, in atypical AD, eg, posterior cortical atrophy, visual dysfunction may be more remarkable than the verbal memory [44,45]. To be more inclusive in the memory assessment, the CNCB also includes the brief visual memory test.

The Preclinical Alzheimer Cognitive Composite (PACC) was recently proposed for the cognitive measurement of preclinical AD [32,46,47]. One significant difference between the CNCB and PACC lies in the test using a controlled learning paradigm. The PACC uses the free and cued selective reminding test (FCSRT) [48], and the CNCB suggests the use of the memory binding test (MBT). Developed by Buschke et al., both tests are different from conventional learning and memory tests [49,50]. Using the controlled learning paradigm, both tests could minimize the utilization of an individual's learning strategies. Previous studies have shown that both tests could distinguish aMCI from healthy controls with relatively high sensitivity and specificity [49,51-54]. However, as proposed by Buschke et al., the MBT is superior to the FCSRT in terms of using two coordinated word lists to assess associative binding [50,55]. Therefore, the MBT may detect early presymptomatic memory impairment indicated by low binding. Moreover, the validation of the Chinese version of the MBT has been completed [56]. Thus, the inclusion of the MBT in the CNCB may be more promising to identify subtle memory impairment.

Compared with the PACC, the CNCB uniquely includes measures for social cognition. Social cognition has recently received increasing attention in cognitive neuroscience. Previous studies have shown that frontotemporal dementia has more impairment in social cognition than Alzheimer disease [57,58]. Individuals with MCI may exhibit dysfunction in a financial capacity, although their daily living activities have not been significantly impaired [59,60]. The impairment in financial capacity may be explained by the deficit in risk judgment and decision-making [61,62]. Several studies suggest social cognition could be a sensitive predictor of MCI and an index to identify dementia from normal aging individuals [63-65]. Therefore, to increase the ecological utility, the experts agreed to include social cognition in the CNCB. It was not surprising that the expert survey exhibited a great variation, because social cognition in AD and MCI has not been well studied. The face-to-face meeting allowed the advisory members to comment and discuss fully when the preliminary research findings of a pilot study were shared.

### Limitations

One critical concern is that only one round expert survey was performed to collect opinions about the neuropsychological tests. The iterative procedure comprising several rounds of enquiry would be more informative. However, the experts may be bound to their experiences in applying the tests locally. As expected, they are not familiar with recently validated tests. To overcome the potential barriers in reaching the consensus through rounds of survey, the modified Delphi method might be an effective alternative. The methodology has been used to address the complex and multi-factorial problems that might not initially meet consensus [66,67]. We applied the modified Delphi method that integrated the face-to-face expert meetings with the online anonymous survey in reaching the consensus. The face-to-face meetings allow better exchange of different perceptions and ideas and reassessment of the list of the cognitive tests. As certain newly validated tests are not commonly used, live discussions on the face-to-face meetings are of particular value. In addition, we use the online expert survey that actually increases the efficiency of the process and secures a response rate greater than 80%.

The Chinese Neuropsychological Consensus Battery (CNCB) is primarily similar to the cognitive package used in the ongoing dementia prevention trials in the US and European countries. It also has its uniqueness in several aspects: the instruments are culturally appropriate and have been validated in Chinese; memory function will be examined using the controlling learning paradigm; and decision-making and emotional recognition will be explored to detect subtle changes in cognitive function ecologically.

As we reached the consensus on the neuropsychological tests that are potentially useful in the detection of MCI and AD, the CN-NORM project has moved to Stage 2, ie, establishing the norms in a nationally representative sample. Several factors, including age, education, gender, household registry and geographic distribution, are taken into consideration. Moreover, the selected neuropsychological tests will be administered to elderly individuals with neurocognitive disorders and psychiatric disorders in clinical settings (Stage 3). The data-driven approach will ultimately be used to recommend the optimal neuropsychological measures for tailoring the early detection of MCI and common subtypes of dementia in community settings and memory clinic practice.

## Additional material

Online Supplementary Document
