# Supplementary material for: Consensus statement on the neurocognitive outcomes for early detection of mild cognitive impairment and Alzheimer dementia from the Chinese Neuropsychological Normative (CN-NORM) Project
Source: J Glob Health. 2019 Dec 16;9(2):020320. doi: 10.7189/jogh.09.020320 (PMC6925962; doi:10.7189/jogh.09.020320)
Supplement: Online Supplementary Document [file jogh-09-020320-s001.pdf]

Appendix S1. The three-step strategy for the literature search

Step 1. Search of the literature was performed using the terms: “Alzheimer* and [*subtype of dementia*] and Cognit* and meta," "Alzheimer* and Frontal dementia and Neuropsychol* and meta," "Alzheimer* and Differentiate and Cognit* tests" and "dementia and Differentiate and Neuropsychol* tests."

Step 2. For the studies published after the published meta-analysis, we researched the references using the terms: "Alzheimer and Frontotemporal Dementia and Neuropsychological tests and Differentia*."

Step 3. Further literature search was based on the published relevant references. All literature research was conducted through PubMed.

Appendix S2. Qualitative literature review of six cognitive domains

2.1 Attention

The literature demonstrated the utility of the trail making test -A, digit span forward is moderate in the detection of MCI and differentiation of AD from other types of dementia [1–3]. Although the performances of the vigilance task (FTD vs. AD) and the test of everyday attention (DLB vs. AD) were different between AD and other dementias, the discriminatory utility was not thoroughly examined [4,5].

2.2 Memory

Free recall, cued recall, and associative learning and recall were three standard categories of memory tests. Most studies have shown immediate and delayed free call of the learning and memory tests (e.g., HVLT, AVLT, CVLT, CERAD word list learning, RBANS list learning, RBANS story memory, WMS logical memory, WMS visual reproduction, and visual route learning test) are powerful to differentiate AD from healthy controls [6–12]. Several studies found that HVLT was not sensitive to detect MCI from healthy controls [13].

Using the cued recall paradigm, the category cued recall test had higher sensitivity and specificity in diagnosing AD [14], and the FCSRT achieved a specificity of 83% for the detection of MCI [15].

In the tests with the associative learning paradigm, most tests were used to differentiate AD from healthy controls [16–18], with the exception of the memory binding test (MBT) [19,20]. Previous studies have demonstrated that the MBT is a promising new memory test for MCI [20,21].

2.3 Executive function

Although many studies have observed the impairment of executive function in AD [1,2,22,23], very few studies have explored how useful executive function could assist in the detection of MCI.

2.4 Visuospatial function

Previous studies have indicated individuals with AD exhibited a significant impairment of visuospatial function measured with the block design, clock drawing test, Rey-Osterrith Complex Figure recall test, and Benton visual retention test, thus indicating their potential for the diagnosis of AD [24]. As newly developed tests, the four mountains test and supermarket trolley test have shown high sensitivity for the detection of MCI [25,26].

2.5 Language

Most language tests have shown a moderate effect (effect size ranging from 0.88 to 1.39) in differentiating AD and FTD [2,3]. Verbal fluency tests are sensitive (sensitivity ranged from 0.87 to 0.91) in distinguishing individuals with dementia from healthy controls [27,28]. The Boston naming test is sensitive to detect MCI individuals who converted to AD [29].

2.6 Social cognition

Previous studies have demonstrated that social cognition tests, e.g., the faux pas recognition, Ekman faces test, and reading the mind in the eye test, could differentiate FTD from healthy controls with a sensitivity greater than 90% or an effect size ranging from 1.38 to 2.28 [2,30–32]. However, the performance on the false belief tasks was only slightly different between AD and FTD [32,33].

Appendix S3. Detailed introduction of the CNCB tests

3.1 Attention

a. digit span — forward: This subtest of the Wechsler memory scale (WMS) is commonly used [1,2]. The Chinese version of the WMS was first validated in 1981. In a multicenter study in Asia, the digit span test was validated with other neurocognitive tests [2,3].

b. Trail making test A: The TMT-A measures the speed of processing. It has been validated in a multicenter study to standardize the neuropsychological assessment in adults in China [2].

c. Flanker test (NIH EXAMINER/Toolbox): The Flanker test measures the effect of conflicting information within a stimulus set [4]. In this task, irrelevant stimuli must be inhibited to respond to a relevant target stimulus. The target is flanked by nontarget stimuli, which correspond to the same directional response as the target (congruent flankers), the opposite response (incongruent flankers), or neither (neutral flankers). In the tests, a directional response (typically left or right) is assigned to a central target stimulus. Various forms of the task are used to measure information processing and selective attention.

3.2 Memory

a. Hopkin's verbal learning test: The HVLT-R measures short- and long-term auditory memory. Each form of the HVLT-R consists of a 12-item wordlist, composed of words from each of the three semantic categories. The validated Chinese version of the HVLT-R has been widely used to assess neurocognitive function in individuals with HIV/AIDS and schizophrenia [5–7]. In addition to the conventional procedure of administering the HVLT test, experts have proposed the addition of a 5-min delayed recall to increase the utility for

MCI screening [8,9].

b. Brief visuospatial memory test: The BVMT-R measures visual-graphic memory. It includes multiple presentations to assess learning and has six alternate forms that can be used in serial evaluations to avoid practice effects. The Chinese version of the test has been validated in a multicenter study to standardize the neuropsychological assessment in adults [10].

c. Logical memory: The task is a subset of the WMS and measures memory for conceptually related verbal information. The validated Chinese version of logic memory has been widely used to measure the episodic memory of Alzheimer’s disease and related cognitive disorders.

To minimize the practice effect for repeated measurements, experts have proposed using the stories from the RBANS as alternatives for the WMS logical memory.

d. Memory binding test: The Memory Binding Test (MBT), initially referred to as the Memory Capacity Test, was developed to assess rational binding using semantic words. Compared with the conventional wordlist learning tests, the MBT uses controlled learning and a cued recall paradigm, which requires all individuals to learn and process the learned items with the same procedure and specific encoding strategy. By assuring the same level of attention to and inducing deep semantic processing of the learned items, it provides an additional measure of associative binding through specific encoding and thus achieves a maximal level of retrieval. Thus, the memory performance could be measured relative to the individual's cognitive performance rather than by comparison to peer performance [11,12]. In several countries, the MBT has been shown to have reasonable convergent validity and high reliability [13,14]. Previous studies suggested the MBT achieved good discriminative validity for distinguishing aMCI from healthy elderly subjects [12]. It could also predict incident aMCI in a longitudinal study [15].

Albert Einstein College of Medicine authorized Prof. Huali Wang to translate and validate the MBT in Chinese under an agreement effective as of December 16, 2016. Dr. Wang's team made linguistic modifications for one category and several words on the list. For example, the category of "State" in English is changed to "Province" (*Sheng, 省*) in Chinese. The items of English names (i.e., '*Paul*' and '*Harry*') are changed to Chinese names [i.e., '*Zhang Tao'* (*张涛*) and '*Wang Yong*' (*王勇*)]. The Chinese version of the MBT has been validated (data submitted).

The short-term visual binding test is an alternative form of the MBT. Previous studies suggested it is free of an educational effect and sensitive to detect very early memory impairment [16–18].

3.3 Executive function

a. Trail making test — B: The task measures the functioning of switching. Several forms of the TMT-B, e.g., color and shape versions, have been validated in Chinese [10,19]. The validated version of the color trail making test has been widely used to measure cognitive function in HIV/AIDS, schizophrenia, dementia and related cognitive impairments. Moreover, a multicenter study has established the norms of the color trail making test in Chinese adults [10].

b. Digit span — backward: The Digit span-backward measures working memory. The test has been validated with the WMS in Chinese and has been widely used in different neuropsychiatric disease research.

c. Digit symbol substitution: The Digit symbol substitution task measures the short-term memory and speed of processing. It consists of nine digit-symbol pairs (e.g., 1/-, 2/┴ ... 7/Λ, 8/X, 9/=) followed by a list of digits. No specific changes were made in the Chinese version. It has been validated with the Wechsler Intelligence Scale.

d. Stroop test: The Stroop test measures the inhibition and speed of processing. It

has been employed in China for more than three decades [20,21]. The test is appropriate for elderly individuals as most of them could read the word indicating the name of colors.

3.4 Language

a. Verbal fluency (animal): The test often raises concerns regarding cultural issues. For example, it remains controversial whether mythical animals (i.e., dragon, Phoenix) should be given credit. In the most recent validation study in Asian countries, the name of ‘dragon' is credited, but not ‘Phoenix' [3]. Because dragon is one of the Chinese Zodiacs, symbolizing the year of the animal, individuals would prefer to grant dragon as a valid name of an animal.

b. Boston naming test: The Chinese version of the Boston naming test has been used in a community-based study on dementia and cognitive impairment in Shanghai [22]. More recently, it is also included in the Chinese version of the NINDS-Canadian Stroke Network (NINDS-CSN) neuropsychological battery for vascular cognitive impairment [23].

3.5 visuospatial function

a. Clock drawing test: Since the introduction into China in the early 2000s, the clock-drawing test has achieved widespread clinical use as a cognitive screening instrument in China [24,25].

Conventionally, an individual is required to draw the face of a clock, put in all of the numbers, and set the hands for ‘10 after 11’. Recent studies reported that the use of the digital CDT (dCDT) yielded a higher diagnostic accuracy for the discrimination of aMCI patients from HCs than the use of the conventional (cCDT) even in aMCI patients with normal cCDT scores [26].

b. Judgment of Line Orientation Test (JLOT): The test is a widely used measure of

visuospatial judgment, particularly in Lewy body disease. The subject is instructed to match several pairs of angled lines with the corresponding lines in a radially arranged 11-line display. An advantage of this test over many clinical tests used to assess visuospatial functioning is that it imposes minimal demands on praxis, mnestic, or executive functions [27]. Studies have suggested that it may be useful to differentiate DLB patients from AD and PCA patients [22,28,29].

d. Visual Object and Space Perception (VOSP) – Silhouettes: The VOSP battery evaluates visuospatial function. In this test, silhouettes (shadows) of animals and objects are shown to the participant, and he/she is instructed to identify them. There are eight silhouettes of animals and seven silhouettes of inanimate objects in the present version. The boards are arranged in ascending order of difficulty, and the test should be terminated after five errors. The revised Chinese version has been validated in a memory clinic sample [30].

3.6 Social cognition

a. Facial Emotion Recognition Test: The Facial emotion recognition Test is frequently used to assess social cognition among different clinical conditions [31]. It typically includes photographs of static faces expressing basic emotions (i.e., happiness, sadness, or fear) and neutral expressions. Subjects view one image at a time and choose the correct emotion label for each face. Many studies have shown that emotion recognition could be helpful in better differentiating between cortical dementias, such as bvFTD and AD [32,33]. The Chinese version uses the faces of Chinese adults [34,35].

b. Iowa Gambling Task: The Iowa Gambling Task (IGT) is one of the most frequently used decision-making tests. Participants are instructed to continuously choose cards from four decks (A, B, C, and D) to make as much money as possible in the game. For every choice made, participants receive a certain amount of cash (rewards); however, some choices also result in the loss of money (penalties). Two decks (A & B) are ultimately risky

(large rewards and large punishments), while C and D are more conservative (small rewards and small punishments). Abnormal performance in this task has been described in different neurodegenerative diseases, such as bvFTD, AD, Parkinson's disease, and Huntington disease. Studies suggest that the IGT could be used to provide complementary information to a frontal test battery, particularly in the early stages of the disease before severe dementia develops [36,37]. Wang et al. revised the amount of cash into Chinese RMB (*yuan*) to adapt it to the local culture. The test is now widely used in the social cognition test in different populations (e.g., young adults and college students) and diseases (e.g., schizophrenia, obsessive-compulsive disorder, and mood disorders) [38–40].

c. Game of Dice task: The Game of Dice Task (GDT) is a gambling task used to assess the capacity to make decisions with explicit rules [41]. This approach provides a means of assessing strategic decision-making and reflective risk-taking behavior [42]. In the Chinese version of this task, participants are instructed to bet on the results of a dice roll. The bets are separated into four possible risk levels with the riskiest level reflecting a potential payout or loss of 1000 *yuan*, then 500 *yuan*, 200 *yuan* and 100 *yuan* for the least risky condition [39,43]. Different abnormal patterns in this task have also been described in different neurodegenerative diseases, such as AD and Parkinson's disease. It may become a diagnostic tool for these diseases [36].

References

[1] Wang J, Zou Y-Z, Cui J-F, Fan H-Z, Chen R, Chen N, et al. [Revision of the Wechsler Memory Scale-Fourth Edition of Chinese version (adult battery)]. Chinese Ment Heal J 2015;29:53–9.

[2] Shen JH, Shen Q, Yu H, Lai J-S, Beaumont JL, Zhang Z, et al. Validation of an Alzheimer’s Disease Assessment Battery in Asian Participants With Mild to Moderate Alzheimer’s Disease. J Gerontol Geriatr Res 2014;3:4. doi:10.4172/2167-7182.1000167.

[3] Shen JHQ, Shen Q, Yu H, Lai J, Beaumont JL, Zhang Z, et al. Validation of an Alzheimer's disease assessment battery in Asian participants with mild to moderate Alzheimer's disease. Am J Neurodegener Dis 2014;3:158–69.
[4] Ehrensperger MM, Taylor KI, Berres M, Foldi NS, Dellenbach M, Bopp I, et al. BrainCheck - A very brief tool to detect incipient cognitive decline: Optimized case-finding combining patient- and informant-based data. Alzheimer's Res Ther 2014;6:1–12. doi:10.1186/s13195-014-0069-y.
[5] Shi J, Tian J, Wei M, Miao Y, Wang Y. The utility of the Hopkins Verbal Learning Test (Chinese version) for screening dementia and mild cognitive impairment in a Chinese population. BMC Neurol 2012;12:136. doi:10.1186/1471-2377-12-136.
[6] Heaton R, Cysique LA, Jin H, Shi C, Yu X, Letendre S, et al. Neurobehavioral effects of human immunodeficiency virus infection among former plasma donors in rural China. J Neurovirol 2008;14:536–49. doi:10.1080/13550280802378880.
[7] Wu JQ, Chen DC, Tan YL, Xiu MH, De Yang F, Soares JC, et al. Cognitive impairments in first-episode drug-naive and chronic medicated schizophrenia: MATRICS consensus cognitive battery in a Chinese Han population. Psychiatry Res 2016;238:196–202. doi:10.1016/j.psychres.2016.02.042.
[8] Zhao Q, Lv Y, Zhou Y, Hong Z, Guo Q. Short-Term Delayed Recall of Auditory Verbal Learning Test Is Equivalent to Long-Term Delayed Recall for Identifying Amnestic Mild Cognitive Impairment. PLoS One 2012;7:e51157. doi:10.1371/journal.pone.0051157.
[9] Xu Y, Chen K, Zhao Q, Li F, Guo Q. Short-term delayed recall of auditory verbal learning test provides equivalent value to long-term delayed recall in predicting MCI clinical outcomes: A longitudinal follow-up study. Appl Neuropsychol Adult 2018;(in press):1–9. doi:10.1080/23279095.2018.1481067.
[10] Yu X, editor. [The Normative Reference of MCCB in China]. Beijing: Peking University Medical Press; 2014.
[11] Buschke H. Rationale of the Memory Binding Test. In: Nilsson L-G, Ohta N, editors. Dement. Mem. 1st ed., Psychology Press; 2014, p. 55–71.
[12] Buschke H, Mowrey WB, Ramratan WS, Zimmerman ME, Loewenstein DA, Katz MJ, et al. Memory binding test distinguishes amnestic mild cognitive impairment and dementia from cognitively normal elderly. Arch Clin Neuropsychol 2017;32:29–39. doi:10.1093/arclin/acw083.
[13] Roman F, Iturry M, Rojas G, Barceló E, Buschke H, Allegri RF. Validation of the Argentine version of the Memory Binding Test (MBT) for Early Detection of Mild Cognitive Impairment. Dement Neuropsychol 2016;10:217–26. doi:10.1590/S1980-5764-2016DN1003008.

[14] Gramunt N, Buschke H, Sánchez-Benavides G, Lipton RB, Peña-Casanova J, Diéguez-Vide F, et al. Reference Data of the Spanish Memory Binding Test in a Midlife Population from the ALFA STUDY (Alzheimer's and Family). J Alzheimer's Dis 2015;48:613–25. doi:10.3233/JAD-150237.
[15] Mowrey WB, Lipton RB, Katz MJ, Ramratan WS, Loewenstein DA, Zimmerman ME, et al. Memory Binding Test Predicts Incident Amnestic Mild Cognitive Impairment. J Alzheimer's Dis 2016;53:1585–95. doi:10.3233/JAD-160291.
[16] Rentz DM, Parra Rodriguez MA, Amariglio R, Stern Y, Sperling R, Ferris S. Promising developments in neuropsychological approaches for the detection of preclinical Alzheimer's disease: A selective review. Alzheimer's Res Ther 2013;5:58. doi:10.1186/alzrt222.
[17] Liang Y, Pertzov Y, Nicholas JM, Henley SMD, Crutch S, Woodward F, et al. Visual short-term memory binding deficit in familial Alzheimer's disease. Cortex 2016;78:150–64. doi:10.1016/j.cortex.2016.01.015.
[18] Parra MA, Saarimäki H, Bastin ME, Londoño AC, Pettit L, Lopera F, et al. Memory binding and white matter integrity in familial Alzheimer's disease. Brain 2015;138:1355–69. doi:10.1093/brain/awv048.
[19] Zhao Q, Guo Q, Li F, Zhou Y, Wang B, Hong Z. The Shape Trail Test : Application of a New Variant of the Trail Making Test. PLoS One 2013;8:e57333. doi:10.1371/journal.pone.0057333.
[20] Hu F, Wang Q, Xu L, Ge L. [Multiple Conflict-driven Cognitive Control Mechanisms of the Flanker, Stroop and Simon Conflict]. J Psychol Sci 2012;35:276–81.
[21] Xu S, Sun H, Wu Z. [Word fluency: Age difference in adulthood and relationship with word memory]. Acta Psychol Sin 1989:337–45.
[22] Xu Y, Chen K, Zhao Q, Guo Q. Comparing the neuropsychological profiles of mild dementia with Lewy bodies and mild Alzheimer's disease. Psychogeriatrics 2018;18:64–71. doi:10.1111/psyg.12293.
[23] Chen X, Wong A, Ye R, Xiao L, Wang Z, Lin Y, et al. Validation of NINDS-CSN neuropsychological battery for vascular cognitive impairment in Chinese stroke patients. BMC Neurol 2015;15:4–9. doi:10.1186/s12883-015-0270-z.
[24] Feng F, Zou Y-Z. [A screening instrument for early dementia: Clock drawing test]. Foreign Med Sci Geriatr 2003;24:13–5.
[25] Meng C, Zhang X, Wang H, Sun H, Liu H, Tang Z, et al. [Clock drawing test for detecting cognitive impairment]. Chinese J Nerv Ment Disord 2004;30:452–4.
[26] Müller S, Preische O, Heymann P, Elbing U, Laske C. Increased diagnostic accuracy of digital vs. conventional clock drawing test for discrimination of patients in the early course of Alzheimer's disease from cognitively healthy individuals. Front Aging

Neurosci 2017;9:1–10. doi:10.3389/fnagi.2017.00101.
[27] Crawford JR, Venneri A, O’Carroll RE. Neuropsychological Assessment of the Elderly. In: Bellack AS, Hersen M, editors. Compr. Clin. Psychol., Elsevier; 1998, p. 133–69. doi:10.1016/B0080-4270(73)00069-9.
[28] Simard M, Van Reekum R, Myran D. Visuospatial impairment in dementia with Lewy bodies and Alzheimer’s disease: A process analysis approach. Int J Geriatr Psychiatry 2003;18:387–91. doi:10.1002/gps.839.
[29] Metzler-Baddeley C, Baddeley RJ, Lovell PG, Laffan A, Jones RW. Visual impairments in dementia with Lewy bodies and posterior cortical atrophy. Neuropsychology 2010;24:35–48. doi:10.1037/a0016834.
[30] Huang L, Chen K, Lin B, Tang L, Zhao Q, Li F, et al. An abbreviated version of Silhouettes test : a brief validated mild cognitive impairment screening tool. Int Psychogeriatrics 2018;(in press):1–8. doi:10.1017/S1041610218001230.
[31] Cotter J, Granger K, Backx R, Hobbs M, Looi CY, Barnett JH. Social cognitive dysfunction as a clinical marker: A systematic review of meta-analyses across 30 clinical conditions. Neurosci Biobehav Rev 2018;84:92–9. doi:10.1016/j.neubiorev.2017.11.014.
[32] Bora E, Velakoulis D, Walterfang M. Meta-Analysis of Facial Emotion Recognition in Behavioral Variant Frontotemporal Dementia. J Geriatr Psychiatry Neurol 2016;29:205–11. doi:10.1177/0891988716640375.
[33] Park S, Kim T, Shin SA, Kim YK, Sohn BK, Park HJ, et al. Behavioral and neuroimaging evidence for facial emotion recognition in elderly Korean adults with mild cognitive impairment, Alzheimer’s disease, and frontotemporal dementia. Front Aging Neurosci 2017;9:1–17. doi:10.3389/fnagi.2017.00389.
[34] Wang K, Hoosain R, Li XS, Zhou JN, Wang CQ, Fu XM, et al. Impaired recognition of fear in a Chinese man with bilateral cingulate and unilateral amygdala damage. Cogn Neuropsychol 2002;19:641–52. doi:10.1080/02643290244000130.
[35] Wang K, Hoosain R, Yang RM, Meng Y, Wang CQ. Impairment of recognition of disgust in Chinese with Huntington’s or Wilson’s disease. Neuropsychologia 2003;41:527–37. doi:10.1016/S0028-3932(02)00171-9.
[36] Gleichgerrcht E, Ibáñez A, Roca M, Torralva T, Manes F. Decision-making cognition in neurodegenerative diseases. Nat Rev Neurol 2010;6:611–23. doi:10.1038/nrneurol.2010.148.
[37] Kloeters S, Bertoux M, O’Callaghan C, Hodges JR, Hornberger M. Money for nothing - Atrophy correlates of gambling decision making in behavioural variant frontotemporal dementia and Alzheimer’s disease. NeuroImage Clin 2013;2:263–72. doi:10.1016/j.nicl.2013.01.011.

[38] Zhang L, Wang K, Zhu C, Yu F, Chen X. Trait anxiety has effect on decision making under ambiguity but not decision making under risk. PLoS One 2015;10:1–21. doi:10.1371/journal.pone.0127189.
[39] Zhang L, Wang X, Zhu Y, Li H, Zhu C, Yu F, et al. Selective impairment of decision making under ambiguity in alexithymia. BMC Psychiatry 2017;17:1–8. doi:10.1186/s12888-017-1537-2.
[40] Ma H, Lv X, Han Y, Zhang F, Ye R, Yu F, et al. Decision-making impairments in patients with Wilson's disease. J Clin Exp Neuropsychol 2013;35:472–9. doi:10.1080/13803395.2013.789486.
[41] Brand M, Fujiwara E, Borsutzky S, Kalbe E, Kessler J, Markowitsch HJ. Decision-making deficits of Korsakoff patients in a new gambling task with explicit rules: Associations with executive functions. Neuropsychology 2005;19:267–77. doi:10.1037/0894-4105.19.3.267.
[42] Pletzer B, M. Ortner T. Neuroimaging supports behavioral personality assessment: Overlapping activations during reflective and impulsive risk taking. Biol Psychol 2016;119:46–53. doi:10.1016/j.biopsycho.2016.06.012.
[43] Dai F, Yuan L, Fang J, Zhang Q, Wang K. Impaired decision making under risky conditions in the acute phase of Graves' thyroitoxicosis. Neurosci Lett 2017;661:1–4. doi:10.1016/j.neulet.2017.08.058.

Table S1. Rating the properties of the candidate tools

Property	Range	Meaning of score
Sensitivity for discrimination of MCI *vs.* HC	0～9	a higher score indicates greater sensitivity
Sensitivity for discrimination of AD *vs.* HC	0～9	a higher score indicates greater sensitivity
Specificity for discrimination of AD *vs.* other types of dementia	0～9	a higher score indicates greater specificity
Specificity for discrimination of AD *vs.* psychiatric disorders in old age	0～9	a higher score indicates greater specificity
Cultural appropriateness	0～10	a higher score indicates greater cultural appropriateness
Reproducibility	0～10	a higher score indicates greater reproducibility
Acceptance by the subject	0～10	a higher score indicates greater acceptance by the subject
Ease of being digitalized	0～10	a higher score indicates easier to be administered with computers

HC: healthy controls; MCI: mild cognitive impairment; AD: Alzheimer's disease

Table S2. Summary of the utility of six cognitive domains in the detection of mild cognitive impairment and dementia

Cognitive domain and test	Main findings		Other comments
	AD/MCI *vs.* HC (sensitivity/specificity)	AD/MCI *vs.* other dementias (size effect)	
**Attention**			
TMT-A	MCI *vs.* NC: 0.78/0.92 [1]	AD *vs.* VaD: 0.2 [2]<br>AD *vs.* FTD: 0.71 [3]	
Digit Span		AD *vs.* VaD: 0.2 [2]<br>AD *vs.* FTD: 0.29 [3]	
Test of Everyday Attention (TEA)			DLB < AD < HC [5]
Vigilance Task			FTD < AD [4]
**Memory**			
Free recall			
HVLT-R	AD *vs.* HC: 0.95/0.93<br>MCI *vs.* HC: 0.69/0.71 [13]		
AVLT	AD *vs.* HC: 0.90/0.91 [10]		
CVLT	MCI *vs.* HC: 0.90/0.84 [6]		
CERAD Word List Learning	AD *vs.* HC: 0.86/0.87 [7]		
RBANS List Learning Total	AD *vs.* HC: 0.82/0.82 [8]		
RBANS Story Memory	AD *vs.* HC: 0.72/0.86 [8]		
WMS Logical Memory	AD *vs.* HC: 0.62/0.61 [9]		
WMS-Visual Reproduction	AD *vs.* HC: 0.90/0.79 [11]		
Visual Route Learning Test	AD *vs.* HC: 0.95/0.94 [12]		
Cued recall / selective reminding			
FCSRT	MCI *vs.* HC: 0.72/0.83 [15]		
Category Cued Recall Test – Cued Recall	AD *vs.* HC: 0.89/0.96 [14]		
Associative Learning			
Visual Association Test	AD *vs.* HC: 0.83/0.91 [16]		
Verbal Paired Associate	AD *vs.* HC: 0.86/1 [17]		
CANTAB-Paired Associate Learning	AD *vs.* HC: 0.68/1 [18]		
WMS Verbal Paired Associates	AD *vs.* HC: 0.82/0.77 [19]		

Visual STM Binding	AD *vs.* HC: 0.77/0.83 [19]		
Memory Binding Test	aMCI & dementia *vs.* HC: 0.84/0.73 [20]		
**Executive function**			
Trail Making Test - B	AD *vs.* HC: 0.83/0.92 [1]		
WCST			most frequently used [22]
Stroop test		AD *vs.* VaD: 0.1 [2]	most sensitive to AD [23]
Go-No-Go			FTD > other groups
**Language**			
Graded Naming Test		AD *vs.* FTD: d = 1.39 [3]	
Word–Picture Matching		AD *vs.* FTD: d = 1.05 [3];	
WAB Spontaneous Speech		AD *vs.* FTD: d = 1.04 [3]	
Picture naming tasks		AD *vs.* FTD: d = 0.99 [3]<br>AD *vs.* VaD: d = -0.40 [2]	
Pyramids and Palm Trees		AD *vs.* FTD: d = 0.9 [3]	
WAB Repetition		AD *vs.* FTD: d = 0.88 [3]	
Boston Naming Test			
Verbal Fluency test	Dementia *vs.* HC: 0.87/0.93 [27]		
Animal Fluency	Dementia *vs.* HC: 0.91/0.34 [28]		
Category fluency	very mild AD *vs.* HC: 0.96/0.88		
**Visuospatial function**			
Block Design			diagnostic potential [24]
Clock Drawing Test			diagnostic potential [24]
Rey-Osterrieth Complex Figure recall			prognostic potential [24]
The Benton visual retention			prognostic potential [24]
Virtual Supermarket test			AD < FTD [26]
The 4 Mountains Test	MCI (positive AD biomarker) *vs.* HC: 1.00/0.78 [25]		
**Social cognition**			
faux pas recognition		FTD *vs.* HC: 2.28	DLB < HC [25]

Pictures from the Ekman database	FTD *vs.* HC: 0.94/1 [30]	Differentiating bvFTD from psychiatric disorders and other neurodegenerative diseases
Emotional Recognition task		Best discriminating AD from VaD [2]
Reading the Mind in the Eyes (RME) test	FTD *vs.* HC:1.38 [32]	DLB < HC
False Belief Tasks	AD *vs.* FTD: -0.1 [32]	

HC: healthy controls; AD: Alzheimer's disease; MCI: mild cognitive impairment; VaD: vascular dementia; FTD: frontotemporal dementia; DLB: Lewy body dementia

References

[1] Wei M, Shi J, Li T, Ni J, Zhang X, Li Y, et al. Diagnostic Accuracy of the Chinese Version of the Trail-Making Test for Screening Cognitive Impairment. J Am Geriatr Soc 2018;66:92–9. doi:10.1111/jgs.15135.

[2] Mathias JL, Burke J. Cognitive Functioning in Alzheimer's and Vascular Dementia: A Meta-Analysis. Neuropsychology 2009;23:411–23. doi:10.1037/a0015384.

[3] Hutchinson AD, Mathias JL. Neuropsychological deficits in frontotemporal dementia and Alzheimer's disease: A meta-analytic review. J Neurol Neurosurg Psychiatry 2007;78:917–28. doi:10.1136/jnnp.2006.100669.

[4] Stopford CL, Thompson JC, Neary D, Richardson AMT, Snowden JS. Working memory, attention, and executive function in Alzheimer's disease and frontotemporal dementia. Cortex 2012;48:429–46. doi:10.1016/j.cortex.2010.12.002.

[5] Calderon J, Perry RJ, Erzinclioglu SW, Berrios GE, Dening TR, Hodges JR. Perception, attention, and working memory are disproportionately impaired in dementia with Lewy bodies compared with Alzheimer's disease. J Neurol Neurosurg Psychiatry 2001;70:157–64. doi:10.1136/jnnp.70.2.157.

[6] Rabin LA, Paré N, Saykin AJ, Brown MJ, Wishart HA, Flashman LA, et al. Differential memory test sensitivity for diagnosing amnestic mild cognitive impairment and predicting conversion to Alzheimer's disease. Aging, Neuropsychol Cogn 2009;16:357–76. doi:10.1080/13825580902825220.

[7] Bertolucci PHF, Okamoto IH, Brucki SMD, Siviero MO, Neto JT, Ramos LR. Applicability of the CERAD neuropsychological battery to Brazilian elderly. Arq Neuropsiquiatr 2001;59:532–6. doi:10.1590/S0004-282X2001000400009.

[8] Duff K, Humphreys Clark JD, O'Bryant SE, Mold JW, Schiffer RB, Sutker PB. Utility of the RBANS in detecting cognitive impairment associated with Alzheimer's disease: Sensitivity, specificity, and positive and negative predictive powers. Arch Clin Neuropsychol 2008;23:603–12. doi:10.1016/j.acn.2008.06.004.

[9] Storandt M, Morris JC. Ascertainment bias in the clinical diagnosis of Alzheimer disease. Arch Neurol 2010;67:1364–9. doi:10.1001/archneurol.2010.272.

[10] Ewers M, Walsh C, Trojanowski JQ, Shaw LM, Petersen RC, Jack CR, et al. Prediction of conversion from mild cognitive impairment to Alzheimer's disease dementia based upon biomarkers and neuropsychological test performance. Neurobiol Aging 2012;33:1203–14. doi:10.1016/j.neurobiolaging.2010.10.019.

[11] Cahn DA, Butters N, Cahn DA, Salmon DP, Wiederholt WC, Corey-Bloom J, et al. Detection of dementia of the Alzheimer type in a population-based sample: Neuropsychological test performance. J Int Neuropsychol Soc 1995;1:252–60. doi:10.1017/S1355617700000242.

[12] Pengas G, Patterson K, Arnold RJ, Bird CM, Burgess N, Nestor PJ. Lost and found: Bespoke memory testing for Alzheimer's disease and semantic dementia. J Alzheimer's Dis 2010;21:1347–65. doi:10.3233/JAD-2010-100654.

[13] Shi J, Tian J, Wei M, Miao Y, Wang Y. The utility of the Hopkins Verbal Learning Test (Chinese version) for screening dementia and mild cognitive impairment in a Chinese population. BMC Neurol 2012;12:136. doi:10.1186/1471-2377-12-136.

[14] Vogel A, Mortensen EL, Gade A, Waldemar G. The Category Cued Recall test in very mild Alzheimer's disease: Discriminative validity and correlation with semantic memory functions. Eur J Neurol 2007;14:102–8. doi:10.1111/j.1468-1331.2006.01568.x.

[15] Lemos R, Simões MR, Santiago B, Santana I. The free and cued selective reminding test: Validation for mild cognitive impairment and Alzheimer's disease. J Neuropsychol 2015;9:242–57. doi:10.1111/jnp.12048.

[16] Lindeboom J, Schmand B, Tulner L, Walstra G, Jonker C. Visual association test to

detect early dementia of the Alzheimer type. J Neurol Neurosurg Psychiatry 2002;73:126–33. doi:10.1136/jnnp.73.2.126.
[17] Lowndes GJ, Saling MM, Ames D, Chiu E, Gonzalez LM, Savage GR. Recall and recognition of verbal paired associates in early Alzheimer's disease. J Int Neuropsychol Soc 2008;14:591–600. doi:10.1017/S1355617708080806.
[18] O'Connell H, Coen R, Kidd N, Warsi M, Chin A-V, Lawlor BA. Early detection of Alzheimer's disease (AD) using the CANTAB paired Associates Learning Test. Int J Geriatr Psychiatry 2004;19:1207–8. doi:10.1002/gps.1180.
[19] Liang Y, Pertzov Y, Nicholas JM, Henley SMD, Crutch S, Woodward F, et al. Visual short-term memory binding deficit in familial Alzheimer's disease. Cortex 2016;78:150–64. doi:10.1016/j.cortex.2016.01.015.
[20] Buschke H, Mowrey WB, Ramratan WS, Zimmerman ME, Loewenstein DA, Katz MJ, et al. Memory binding test distinguishes amnestic mild cognitive impairment and dementia from cognitively normal elderly. Arch Clin Neuropsychol 2017;32:29–39. doi:10.1093/arclin/acw083.
[21] Mowrey WB, Lipton RB, Katz MJ, Ramratan WS, Loewenstein DA, Zimmerman ME, et al. Memory Binding Test Predicts Incident Dementia: Results from the Einstein Aging Study. J Alzheimer's Dis 2018;62:293–304. doi:10.3233/JAD-170714.
[22] de Assis Faria C, Alves HVD, Charchat-Fichman H. The most frequently used tests for assessing executive functions in aging. Dement Neuropsychol 2015;9:149–55. doi:10.1590/1980-57642015DN92000009.
[23] Costa A, Bak T, Caffarra P, Caltagirone C, Ceccaldi M, Collette F, et al. The need for harmonisation and innovation of neuropsychological assessment in neurodegenerative dementias in Europe: Consensus document of the Joint Program for Neurodegenerative Diseases Working Group. Alzheimer's Res Ther 2017;9:27. doi:10.1186/s13195-017-0254-x.
[24] Salimi S, Irish M, Foxe D, Hodges JR, Piguet O, Burrell JR. Can visuospatial measures improve the diagnosis of Alzheimer's disease? Alzheimer's Dement Diagnosis, Assess Dis Monit 2018;10:66–74. doi:10.1016/j.dadm.2017.10.004.
[25] Chan D, Gallaher LM, Moodley K, Minati L, Burgess N, Hartley T. The 4 Mountains Test: A Short Test of Spatial Memory with High Sensitivity for the Diagnosis of Pre-dementia Alzheimer's Disease. J Vis Exp 2016;116:e54454. doi:10.3791/54454.
[26] Tu S, Spiers HJ, Hodges JR, Piguet O, Hornberger M. Egocentric versus Allocentric Spatial Memory in Behavioral Variant Frontotemporal Dementia and Alzheimer's Disease. J Alzheimer's Dis 2017;59:883–92. doi:10.3233/JAD-160592.
[27] Mok EHL, Lam LCW, Chiu HFK. Category Verbal Fluency Test Performance in Chinese Elderly with Alzheimer's Disease. Dement Geriatr Cogn Disord 2004;18:120–4. doi:10.1159/000079190.
[28] Grober E, Hall C, McGinn M, Nicholis T, Stanford S, Ehrlich A, et al. Neuropsychological strategies for detecting early dementia. J Int Neuropsychol Soc 2008;14:130–42. doi:10.1017/S1355617708080156.
[29] Calero MD, Arnedo ML, Navarro E, Ruiz-Pedrosa M, Carnero C. Usefulness of a 15-item version of the Boston Naming Test in neuropsychological assessment of low-educational elders with dementia. J Gerontol Psychol Sci 2002;57B:187–91.
[30] Diehl-Schmid J, Pohl C, Ruprecht C, Wagenpfeil S, Foerstl H, Kurz A. The Ekman 60 Faces Test as a diagnostic instrument in frontotemporal dementia. Arch Clin Neuropsychol 2007;22:459–64. doi:10.1016/j.acn.2007.01.024.
[31] Bora E, Velakoulis D, Walterfang M. Meta-Analysis of Facial Emotion Recognition in Behavioral Variant Frontotemporal Dementia. J Geriatr Psychiatry Neurol 2016;29:205–11. doi:10.1177/0891988716640375.
[32] Bora E, Walterfang M, Velakoulis D. Theory of mind in behavioural-variant frontotemporal dementia and Alzheimer's disease: A meta-analysis. J Neurol Neurosurg Psychiatry 2015;86:714–9. doi:10.1136/jnnp-2014-309445.
[33] Gregory C, Lough S, Stone V, Erzinclioglu S, Martin L, Baron-Cohen S, et al. Theory of mind in patients with frontal variant frontotemporal dementia and Alzheimer's

disease: theoretical and practical implications. Brain 2002;125:752–64. doi:10.1093/brain/awf079.
